# Michael acceptor molecules in natural products and their mechanism of action

**DOI:** 10.3389/fphar.2022.1033003

**Published:** 2022-11-02

**Authors:** Song-Ting Liang, Chu Chen, Rui-Xin Chen, Rui Li, Wen-Li Chen, Gui-Hua Jiang, Lei-Lei Du

**Affiliations:** ^1^ State Key Laboratory of Southwestern Chinese Medicine Resources, School of Pharmacy, Chengdu University of Traditional Chinese Medicine, Chengdu, China; ^2^ Sichuan Provincial Key Laboratory of Quality and Innovation Research of Chinese Materia Medica, Sichuan Academy of Chinese Medicine Sciences, Chengdu, China; ^3^ School of Ethnic Medicine, Chengdu University of Traditional Chinese Medicine, Chengdu, China

**Keywords:** Michael acceptor molecules, bioactive plant components, Keap1-Nrf2-ARE, NF-κB, covalent binding

## Abstract

**Purpose:** Michael receptor molecules derived from plants are biologically active due to electrophilic groups in their structure. They can target nucleophilic residues on disease-related proteins, with significant therapeutic effects and low toxicity for many diseases. They provide a good option for relevant disease treatment. The aim of this study is to summarize the existing MAMs and their applications, and lay a foundation for the application of Michael receptor molecules in life science in the future.

**Methods:** This review summarizes the published studies on Michael receptor molecules isolated from plants in literature databases such as CNKI, Wanfang Data, PubMed, Web of Science, ScienceDirect, and Wiley. Latin names of plants were verified through https://www.iplant.cn/. All relevant compound structures were verified through PubChem and literature, and illustrated with ChemDraw 20.0.

**Result:** A total of 50 Michael receptor molecules derived from various plants were discussed. It was found that these compounds have similar pharmacological potential, most of them play a role through the Keap1-Nrf2-ARE pathway and the NF-κB pathway, and have biological activities such as antioxidant and anti-inflammatory. They can be used to treat inflammatory diseases and tumors.

**Conclusion:** The Michael receptor molecule has electrophilicity due to its unsaturated aldehyde ketone structure, which can combine with nucleophilic residues on the protein to form complexes and activate or inhibit the protein pathway to play a physiological role. Michael receptor molecules can regulate the Keap1-Nrf2-ARE pathway and the NF-κB pathway. Michael receptor molecules can be used to treat diseases such as inflammation, cancer, oxidative stress, *etc.*

## 1 Introduction

**FIGURE 1:**
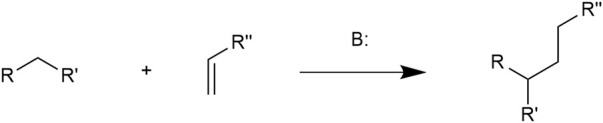
In which the R and R′ substituents on the nucleophile (a Michael donor) are electron-withdrawing groups such as acyl and nitrile and R″ is a nucleophilic group.

The Michael addition reaction (MAR), firstly discovered by Arthur Michael in 1887, is the 1,4-nucleophilic addition of an electron-donating carbanion and an electron-accepting double bond linking to a carbonyl group (Xing et al., 2016). This reaction is catalyzed by a base. Compounds containing α, β-unsaturated bonds and electron withdrawing groups such as carbonyl and ester groups are known as Michael acceptor molecules (MAMs). MAR is in nature a conjugate addition of C-C bond formation. The general MAR formula is illustrated in Figure 1 (Fotouhi et al., 2018).

In the past decades, researchers connected α, β-unsaturated carbonyl compounds with biological processes and revealed some attractive physiological activities of MAMs. The active α, β-unsaturated groups make MAMs susceptible to react with nucleophilic groups. Under physiological conditions, potential nucleophiles that can undergo Michael addition include thiols and amines, which have a ubiquitous presence as small molecules or on proteins (Hearn et al., 2021). For example, cysteine (Cys), homocysteine (Hcy) and glutathione (GSH) are widespread biothiols, while human serum albumin (HAS), and immunoglobulin G (IgG) contain substantial reactive amines. Through MAR, MAMs may change the biological conformation of proteins or enzymes containing nucleophilic groups, thereby inducing consequent physiological changes.

Several MAMs have been developed into drugs and approved for a variety of indications. Dimethyl Fumarate, registered for the treatment of relapsing forms of Multiple Sclerosis and psoriasis, exerts inhibiting effects against inflammation, neurodegeneration and toxic OS through activation of the nuclear factor erythroid 2-related factor 2 (Nrf2) transcriptional pathway as well as interaction with the anti-inflammatory hydroxycarboxylic acid receptor 2 (HCAR2) (Kourakis et al., 2020). Osimertinib, developed to treat drug-resistant non-small cell lung cancer, can inhibit epidermal growth factor receptor (EGFR) tyrosine kinase by MAR with its Cys797 residues on the site reactive to adenosine triphosphate (ATP) (Dong et al., 2018). Acalabrutinib, approved for Relapsed or Refractory Mantle-Cell Lymphoma, is an inhibitor of Bruton tyrosine kinase (BTK) acting by MAR with the thiol in Cys481 (Patel et al., 2017). Also, many MAMs under studies draw increasing attention owing to their outstanding effects. Itaconate exhibits great anti-inflammatory and anti-bacterial activity through inhibiting the key glycolytic enzymes fructose-bisphosphate aldolase A (ALDOA) and lactate dehydrogenase A (LDHA) (Qin et al., 2019). A recently research reported new compound NU6300 can inhibit cyclin-dependent kinases 2 (CDK2) by MAR with the amine in Lys89 (Dong et al., 2018).

**FIGURE 2:**
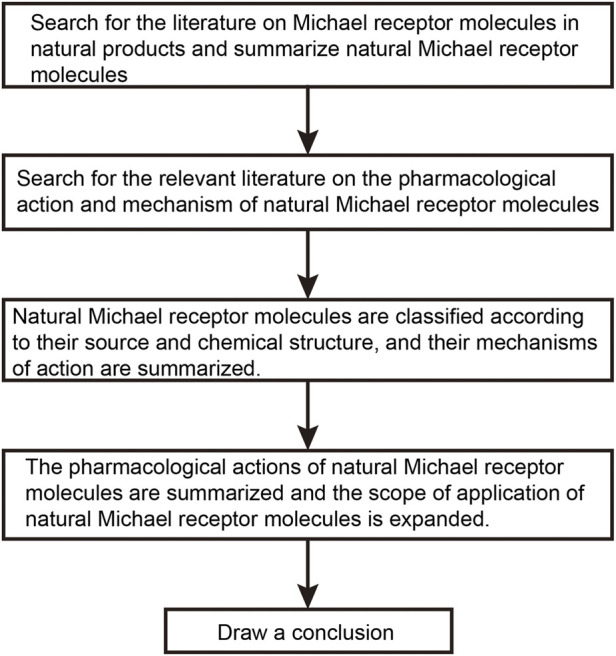
The frame of the article.

MAMs as natural products have been widely discovered in plants, animals and prokaryotic organisms. Natural products are main resource of traditional medicines around the world and account for over 30% of modern chemical drugs. It has long been recognized that natural product structures have the characteristics of high chemical diversity, biochemical specificity and other molecular properties that make them useful as lead structures for drug development (Koehn and Carter, 2005). Thus, structure-function elucidation of natural MAMs may provide inspiration for the development of innovative drugs or candidates. In this work, we provide a summary of reported natural MAMs, as well as their species sources and biological activities. The frame of the article is shown in Figure 2.

## 2 Materials and methods

Using “Michael acceptor molecule,” “Michael reaction molecule,” “Michael molecule,” “Michael reaction and plant,” “α,β-unsaturated compound and plant,” “α,β-unsaturated compound and Michael reaction,” “α,β-unsaturated ketones and plants” as search terms, we searched CNKI, Wanfang Data, PubMed, Web of science, ScienceDirect, and Wiley electronic databases for articles. Articles related to MAMs isolated from natural products were included, and articles related to molecular design were excluded. Summarize the MAMs in the retrieved articles. By searching for relevant articles with the search terms “this compound and α,β-unsaturated compounds”, “this compound and Michael reaction”, “this compound and glutathione” and “this compound and cysteine”, retain the literature whose pharmacological effects may be related to the electrophilic group of MAMs, duplicate literatures and other literatures with low relevance are excluded. A total of 50 MAMs and 72 related literatures were retrieved.

## 3 Results

### 3.1 Michael acceptors in nature and their chemical structures

**FIGURE 3:**
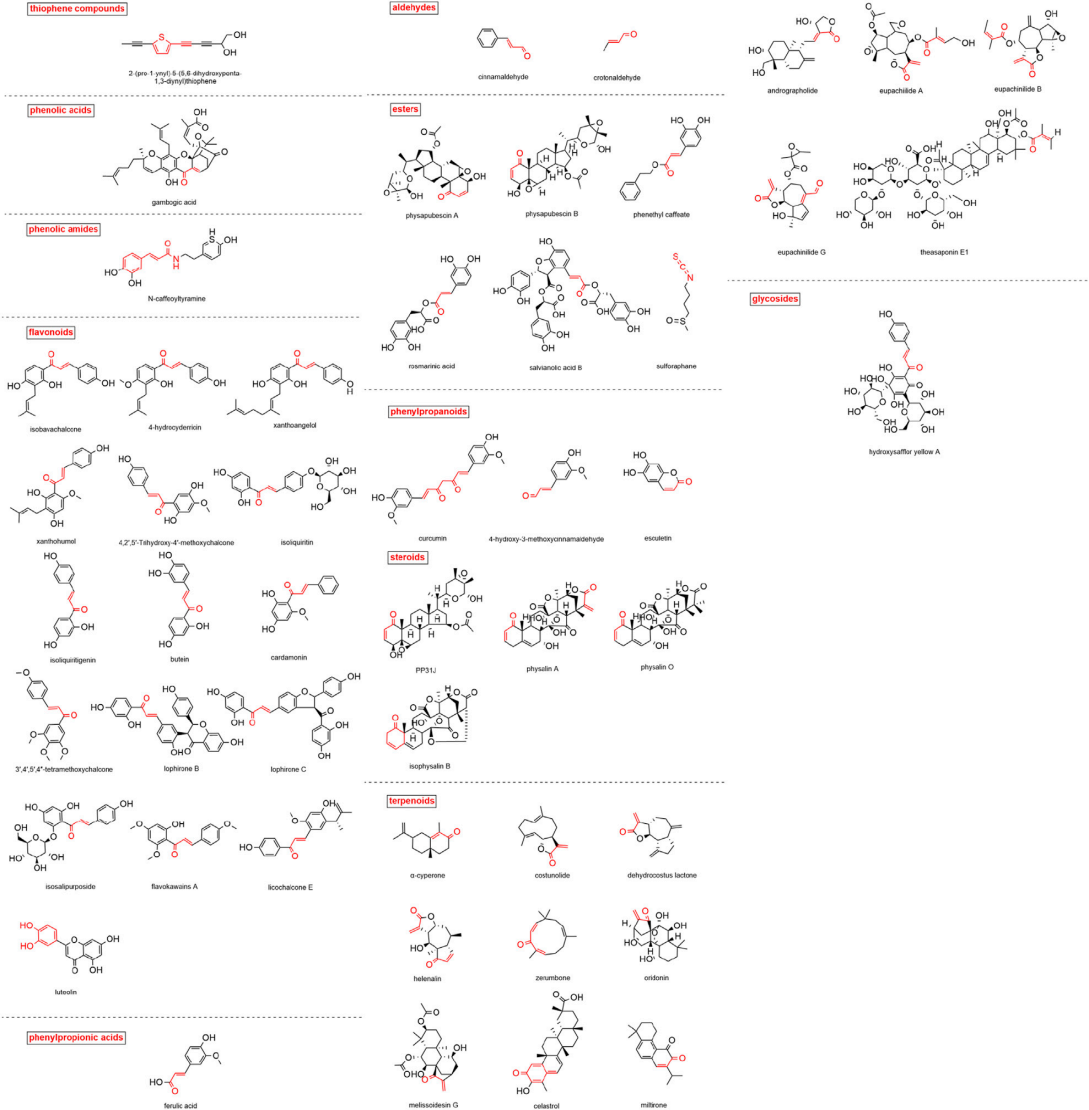
Structure of natural MAMs (Michael reaction active sites are indicated in red).

A total of 50 MAMs in nature were summarized in this study. The classification, sources and research references of these compounds are listed in Table 1. The structures of these compounds are shown in Figure 3.

**TABLE 1:** MAMs from different sources in nature, the source and classification of these MAMs are shown in the table.

The major types of the compounds	MAMs	The sources of compounds	References
thiophene compounds	2-(pro-1-ynyl)-5-(5,6-dihydroxypenta-1,3-diynyl)-thiophene	*Echinops grijsii* Hance	Zhang et al. (2010)
phenolic acids	gambogic acid	*Garcinia hanburyi* Hook. f.	Wang et al. (2015a)
phenolic amides	N-caffeoyltyramine	*Lycium chinense* Mill. or *Lycium barbarum* L.	Xie et al. (2014)
flavonoids	isobavachalcone	*Angelica keiskei* Koidz., *Cullen corylifolium* (Linnaeus) Medikus	Luo et al. (2012)
	4-hydrocyderricin	*A. keiskei* Koidz.	Luo et al. (2012)
	xanthoangelol	*A. keiskei* Koidz.	Luo et al. (2012)
	xanthohumol	*Humulus lupulus* L.	Yao et al. (2015)
	4,2′,5′-Trihydroxy-4′-methoxychalcone	*Dalbergia odorifera* T. Chen	Lee et al. (2013)
	isoliquiritin	*Glycyrrhiza uralensis* Fisch.	Wang et al. (2015b)
	isoliquiritigenin	*G. uralensis* Fisch.	Wang et al. (2015b)
	butein	*Toxicodendron vernicifluum* (Stokes) F. A. Barkl.	Ma et al. (2009)
	cardamonin	*Alpinia hainanensis* K. Schum.	Peng et al. (2017)
	3′,4′,5′,4″-tetramethoxychalcone	*Spatholobus suberectus* Dunn	Peng et al. (2016)
	lophirone B	*Lophira alata* Banks ex C. F. Gaertn.	Ajiboye et al. (2014)
	lophirone C	*L. alata* Banks ex C. F. Gaertn.	Ajiboye et al. (2014)
	isosalipurposide	*Corylopsis coreana* Uyeki	Han et al. (2015)
	flavokawains A	*Piper methysticum* G. Forst.	Pinner et al. (2016)
	licochalcone E	*G. inflata* Batal.	Kim et al. (2012)
	luteolin	*Prunella vulgaris* L.	Liu (2020)
phenylpropionic acids	ferulic acid	*Ferula sinkiangensis* K. M. Shen	Doss et al. (2016)
aldehydes	cinnamaldehyde	*Cinnamomum cassia* Presl	Brackman et al. (2011)
	crotonaldehyde	*Croton tiglium* L.	Lee et al. (2011b)
esters	physapubescin A	*Physalis philadelphica* Lamarck	Ji et al. (2013)
	physapubescin B	*P. philadelphica* Lamarck	Ji et al. (2013)
	phenethyl caffeate	*Apis cerana* Fabricius or *Apis mellifera* Linnaeus	Balogun et al. (2003)
	rosmarinic acid	*Perilla frutescens* (L.) Britt., *Salvia miltiorrhiza* Bunge	Narayan et al. (2011)
	salvianolic acid B	*S. miltiorrhiza* Bunge	Li et al. (2021)
	sulforaphane	*Brassicaceae* Burnett	Huang et al. (2019)
phenylpropanoids	curcumin	*Curcuma longa* L.	Narayan et al. (2011)
	4-hydroxy-3-methoxycinnamaldehyde	*Pinaceae Spreng.* ex F. Rudolphi	Akber et al. (2015)
	esculetin	*Fraxinus chinensis* Roxb.	Arora et al. (2016)
steroids	PP31J	*P. philadelphica* Lamarck	Xu et al. (2014)
	physalin A	*Alkekengi officinarum* var. *Franchetii* (Mast.) R. J. Wang	Ji et al. (2012)
	physalin O	*A. officinarum* var. *Franchetii.*	Ji et al. (2012)
	isophysalin B	*P. minima* L.	Men et al. (2016)
terpenoids	α-cyperone	*Cyperus rotundus* L.	Ye and Gao, (2008)
	costunolide	*Aucklandia costus* Falc.	Liu et al. (2019a)
	dehydrocostus lactone	*A. costus* Falc.	Lee et al. (2020)
	helenalin	*Arnica montana* L.	Fang et al. (2021)
	zerumbone	*Zingiber zerumbet* (L.) Roscose ex Smith	Wang et al. (2019)
	oridonin	*Isodon serra* (Maximowicz) Kudo	Wang et al. (2017)
	melissoidesin G	*I.* (Benth.) Kudo	Yu (2007)
	celastrol	*Tripterygium wilfordii* Hook. f.	Divya et al. (2016)
	miltirone	*S. miltiorrhiza* Bunge	Ma et al. (2009)
	andrographolide	*Andrographis paniculata* (Burm. f.) Nees	Yuan et al. (2012)
	eupachiilide A	*Eupatorium chinense* L.	Jiang et al. (2020)
	eupachinilide B	*E. chinense* L.	Jiang et al. (2020)
	eupachinilide G	*E. chinense* L.	Jiang et al. (2020)
	theasaponin E1	*Camellia sinensis* (L.) O. Ktze.	Li et al. (2013)
glycosides	hydroxysafflor yellow A	*Carthamus tinctorius* L.	Miao (2019)

### 3.2 Pharmacological action and mechanism of Michael acceptor molecules

#### 3.2.1 Regulate Keap1-Nrf2-ARE pathway

##### 3.2.1.1 Components of the signal pathway

**FIGURE 4:**
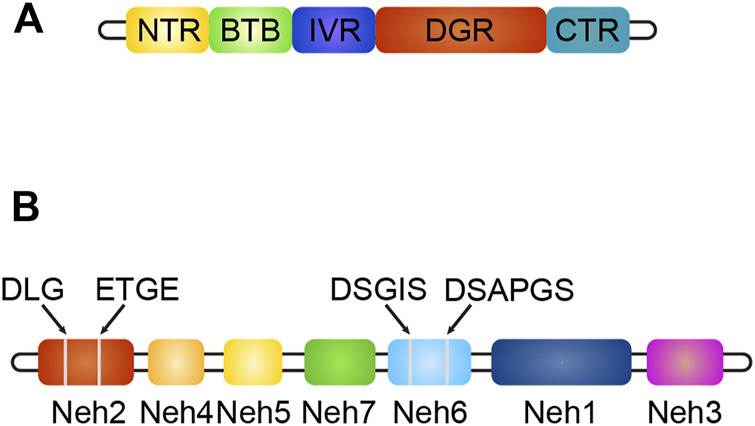
**(A)** The structure of Keap1, different colors indicate different domains. **(B)** The structure of Nrf2, different colors indicate different domains, gray line segments indicate motifs.

Keap1 (Kelch-like ECH-associated protein-1) is a substrate adaptor protein of the ubiquitin ligase E3 (Cul3) complex, which can be connected with Cul3 and Rbx1 to form an E3 ubiquitin ligase complex (Keap1-Cul3-Rbx1), and then regulates Nrf2 (Yao et al., 2019). It contains five domains: NTR, BTB, IVR, DGR, and CTR (Yao et al., 2019). The structure of Keap1 is shown in Figure 4. The BTB domain is responsible for binding Cul3 to form the Keap1-Cul3 ligase complex, mediating ubiquitination of Nrf2 and degradation of proteasome, and promoting the formation of Keap1 dimers (Li, 2018). The DGR domain interacts with the ETGE and DLG motifs of Nrf2, linking Nrf2 to the E3 complex and degrading Nrf2 by ubiquitination (Li, 2018). The IVR domains connects the BTB and DGR domains, is rich in cysteine residues, contains substrate ligand of the ubiquitin ligase complex, and is a site of electrophilic or oxide reaction (Liu et al., 2015). Conformational change following electrophilic stimulation dissociates Keap1 from Nrf2 (Liu et al., 2015; Li, 2018).

Nuclear factor erythroid two related factor 2 (Nrf2) is composed of seven domains, namely Neh1-Neh7 (Li, 2018). The structure of Nrf2 is shown in Figure 4. The functions of each domain are as follows: the Neh1 domain contains the CNC-bZIP region, which is in charge of forming a heterodimer with the small Maf (SMAF) protein and binding to antioxidant response element (ARE) on DNA; the Neh2 domain negatively regulates Nrf2 by combining Keap1 with ETGE and DLG motifs to stabilize it in the cytoplasm; the Neh3 domain can connect with CHD6 to participate in the activation of the ARE gene and is the transactivation domain of Nrf2; the Neh4 domain and Neh5 domain bind to CREB binding protein (CBP) and/or receptor-associated co-activator three when Nrf2 binds to SMAF protein to form a dimer and binds to ARE, participating in initiating downstream gene transcription (Hayes and Dinkova-Kostova, 2014). They are the transactivation domain of Nrf2; the Neh6 domain mediates ubiquitination degradation of Nrf2 through DSGIS and DSAPGS motifs; the Neh7 domain mediates interaction with retinol receptors to inhibit Nrf2 activation (Hayes and Dinkova-Kostova, 2014).

ARE is a DNA enhancer sequence with cis-acting (Huang et al., 2021)

##### 3.2.1.2 Physiological activation process

**FIGURE 5:**
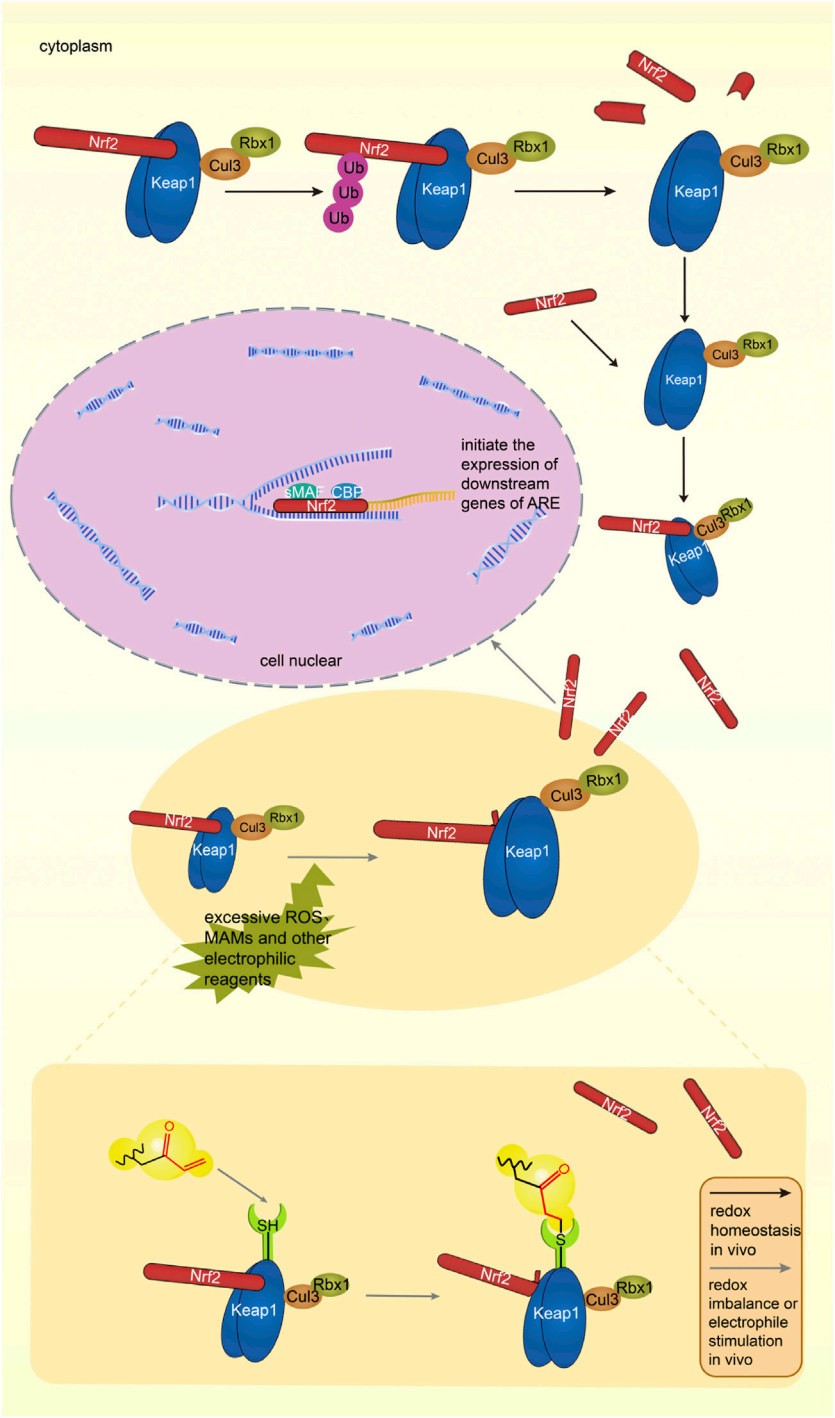
The Keap1-Nrf2-ARE pathway under physiological condition, Keap1 and Nrf2 form a complex in the cytoplasm, and Keap1 degrades Nrf2 through the proteasome pathway. The Keap1-Nrf2-ARE pathway in an activated state. Excessive ROS and electrophiles in cells lead to oxidative stress in the body. Nrf2 dissociates from Keap1, enters the nucleus, binds to ARE with sMAF and CBP, initiates the transcription of antioxidant genes, and induces the expression of antioxidant proteins. MAMs can target cysteine residues of Keap1, activate the Keap1-Nrf2-ARE pathway, and induce the expression of antioxidant proteins. Black arrows indicate normal physiological processes in the body. Gray arrows indicate the physiological process after MAMs react with thiol groups.

Under physiological conditions, the body’s oxidative - antioxidant system maintains in balance. Nrf2 binds to the dimer of Keap1 and is inactive. The complex is subsequently degraded by the ubiquitination-proteasome pathway. Figure 5 shows the Keap1-Nrf2-ARE pathway under physiological conditions *in vivo* (Yao et al., 2019).

When stimulated by the external environment, the oxidative and antioxidant effects are out of balance, which is called oxidative stress (OS). A lot of oxidative intermediate products are produced, such as reactive oxygen species (ROS), which can cause pathological changes in cells and tissues (Wardyn et al., 2015). Specific cysteine residues in Keap1 are modified, the conformation of the Keap1-Cul3 ubiquitin ligase complex is changed, the DLG motif of Nrf2 is dissociated from Keap1, and only the ETGE motif is attached to Keap1, making Keap1 remain saturated (Yao et al., 2019). The newly produced Nrf2 accumulates, enters the nucleus, forms a dimer with the SMAF protein, and cooperates with the transcription factor CBP to bind the relevant sequence on the ARE, activate the pathway, initiate the transcription of downstream target genes, and improve the body’s antioxidant capacity (Huang et al., 2021).

The target genes regulated by the Nrf2-ARE pathway include antioxidant enzymes, detoxification enzymes, DNA repair enzymes and molecular chaperone protein, *etc.* (Wang and Jing, 2016), up to hundreds. Antioxidant enzymes include NAD(P)H: quinone oxidoreductase 1 (NQO1), glutathione-S-transferase (GST), heme oxygenase (HO-1), catalase, superoxide dismutase (SOD), γ-glutamylcysteine synthetase, glutathione reductase, peroxide redoxins Ⅰ, Ⅱ and Ⅲ, thioredoxin, *etc.* These proteins play important roles in physiological process. The Keap1-Nrf2-ARE pathway is closely related to diseases such as central nervous system disease, cardiovascular system disease, liver disease (Cao et al., 2015), lung disease, kidney disease, tumors, etc. (Liu et al., 2015).

##### 3.2.1.3 Activation process of Keap1-Nrf2-ARE signaling pathway stimulated by Michael receptor molecules

Activation process of Keap1-Nrf2-ARE signaling pathway stimulated by MAMs is shown in Figure 5. *In vivo*, MAMs react with cysteine residues of Keap1 to inhibit the complete release of Nrf2 from Keap1, and the continuously generated Nrf2 enters the nucleus and initiates ARE transcription (Huang et al., 2021).

##### 3.2.1.4 Michael receptor molecules that regulate the Keap1-Nrf2-ARE signaling pathway and glutathione

The PYDDT [2-(pro-1-ynyl)-5-(5,6-dihydroxypenta-1,3-diynyl)-thiophene] can bind to intracellular GSH and rapidly down-regulate GSH content *in vitro* experiments (Zhang et al., 2010). Down-regulated GSH/GSSG ratio causes glutathionylation of Keap1, which dissociates from Nrf2 and activates the ARE gene to induce phase II metabolic enzyme transcription and translation. This effect can be inhibited by adding GSH, which may be another activation mechanism of MAMs (Zhang et al., 2010).

Isobavachalcone, 4-hydrocyderricin and xanthoangelol can combine with sulfhydryl group of GSH through α, β-unsaturated carbonyl groups (Luo et al., 2012). They can also induce NQO1, accelerate the clearance of DPPH free radicals, inhibit the generation of α-glucosidase, prevent pancreatic *β* cell secretion dysfunction in diabetic patients, and inhibit the development of diabetes (Luo et al., 2012). Oridonin and ponicidin are both cytotoxic to tumor cells. They have the same basic structure except for three different substituents. The MAR activity of oridonin is higher than that of ponicidin (Liu et al., 2014), and the antitumor activity is also better (Wang et al., 2017). It is speculated that the active center may be the common α, β-unsaturated ketone structure or the methylene cyclopentanone structure. Multitone and its derivatives contain α, β, γ, δ-unsaturated ketone structure and cross conjugated olefin structure, which can undergo MAR with GSH, leading to Nrf2 release and inducing quinone reductase (QR) expression by binding to Keap1 (Ma et al., 2009).

Ji et al. (2013) found that physapubescin A and physapubescin B could induce QR and react with GSH. They have stronger inductive activities than constructive similar compounds without α, β-unsaturated ketone structures, suggesting that the MAMs structure plays an important role in the induction of QR (Ji et al., 2013).

##### 3.2.1.5 Michael acceptor molecules that regulate Keap1-Nrf2-ARE signaling pathway and its downstream genes

Isoliquiritin (ILQ) and isoliquiritigenin (ILG) can increase the nuclear translocation of Nrf2 and induce the expression of antioxidant enzymes by inhibiting Keap1. It was found that the long conjugate structure of ILQ and ILG may induce the expression of HO-1 (Wang R. et al., 2015). Butein can induce nuclear translocation of Nrf2, increase the expression of HO-1 in adipocytes, and inhibit the production of adipocyte and inflammatory cytokines by activating p38 MAPK and degrading Keap1 (Wang et al., 2017). Phenethyl caffeate contains electrophilic α, β-unsaturated carbonyl groups, with effect of inducing ARE equivalent to CUR (Balogun et al., 2003). It promotes the dissociation of the Keap1-Nrf2 complex, upregulates the expression of HO-1 *via* the Nrf2-ARE pathway, and induces the expression of HO-1 by activating p38MAPK in renal epithelial cells (Balogun et al., 2003).

*In vitro*, Zerumbone can increase levels of PARK7, Nrf2 and HO-1, improve the viability of human neuroblastoma cells treated with 1-methyl-4-phenylpyridinium and reduce the ROS content and the number of apoptosis (Cao et al., 2018). Andrographolide can activate the Keap1-Nrf2 pathway and induce the expression of QR by alkylating Cys77, Cys151, Cys273, and Cys368 of Keap1 (Cao et al., 2018). Ning Li (Li et al., 2013) separated seven components from the total saponins of tea seeds. They have the same basic structure, but the substituents at four positions are different. Among them, theasaponin E1 is cytotoxic to human tumor cell lines K562 and HL60, in which angelica acyl group is an essential group and has obvious QR-inducing activity (Li et al., 2013). Angelica acyl and acetyl groups play important roles in the induction of QR (Li et al., 2013). QR can protect cells from oxidation of ROS and electrophilic substances generated in the metabolism of toxic substances and reduce cancer risk.

(10E,12Z)-9-oxo-10,12-octadecadienoic acid and 13-oxo-9(Z),11(E)-octadecadiene acid in damaged Arabidopsis leaves can induce GST1 gene expression and cause cell death through cell damage pathways (Vollenweider et al., 2000). The active site is α, β-unsaturated carbonyl structure, which belongs to the molecular structure of MAMs.

Salvianolic acid B can inhibit LPS-induced acute lung injury by reducing the expression of Keap1, increasing the level of Nrf2 and inducing the expression of NQO1 and HO-1 (Li et al., 2021). Xanthohumol (Xn) is a flavonoid that contains an isoprenyl group. Yao et al. (2015) proved that Xn is an activator of the Nrf2-ARE pathway in neuronal cells, and can upregulate the expression of NQO1 and HO-1. It indirectly promotes the combination of the Nrf2-ARE by promoting the transfer of Nrf2 from the cytoplasm to the nucleus, and the active center is the α, β-unsaturated ketone. The isoprenyl group in Xn can increase its lipophilicity and promote the neuroprotective effect of Xn due to its ability to across the blood-brain barrier (Yao et al., 2015). Licochalcone E (Lico-E) can also activate the Nrf2-ARE pathway, upregulate the expression of NQO1 and HO-1, inhibit LPS-induced inflammatory response in microglia, and protect dopaminergic nerve degeneration in the substantia nigra striatum of mice (Kim et al., 2012). It can be used for the treatment of Parkinson’s disease. This series of reactions may be caused by the α, β-unsaturated carbonyl in Lico-E (Kim et al., 2012).

Celastrol has a variety of biological activities. It may induce Nrf2 dissociation, increase the expression of phase II enzymes such as HO-1, GSTs and NQO1 by combining with nucleophiles (such as Keap1) in cells and can be used to treat pulmonary fibrosis (Divya et al., 2016). Luteolin can downregulate Keap1, activate the Nrf2-ARE pathway, increase the expression of γ-glutamylcysteine synthetase, HO-1, and NQO1, and reduce the level of OS caused by hypoxia-reoxygenation, thereby inhibiting cardiomyocyte apoptosis (Liu, 2020).

*L. alata* Banks ex C.F.Gaertn., which grows in the tropical rain forests of Africa and is traditionally used to treat toothaches, liver infections, *etc.* The lophirone B and C in its stem bark extract can target and inhibit the action of Keap1 by chemically modifying the specific cysteine residue of Keap1, induce Nrf2 nuclear translocation, promote the expression of downstream NQO1, HO-1, UGT, SOD, GST, and EPH, and enhance cellular antioxidant capacity and liver detoxification enzyme activity (Ajiboye et al., 2014).

Esculetin (ESC) can bind to Keap1 to promote the dissociation and the accumulation of Nrf2 in the nucleus, thereby inhibiting the growth of pancreatic cancer cells (Arora et al., 2016). Cardamonin can modify the cysteine residues of Keap1 through α, β-unsaturated carbonyl structure to form a covalent adduct, promote nuclear translocation of Nrf2, and initiate the expression of downstream genes of ARE (Peng et al., 2017). Cardamonin can penetrate the blood-brain barrier to exert a neuroprotective effect and can be used as a candidate drug for the prevention of OS-mediated neurodegenerative diseases (Peng et al., 2017).

#### 3.2.2 Inhibit the NF-κB pathway

##### 3.2.2.1 Components of the signal pathway

In mammals, there are five types of NF-κB protein family: p65, p50, p52, RelB, and c-Rel (Liu et al., 2008). In the resting state, they are in a dimerized state and bind to its inhibitory protein IκB in the cytoplasm (Li, 2009). After cells are stimulated by the external environment, IκB kinase (IKK) phosphorylates IκB (Li, 2009). IκB is degraded after ubiquitination and releases NF-κB (Li, 2009). NF-κB enters the nucleus and binds to the corresponding binding site to initiate gene transcription (Liu et al., 2008). The transcribed genes include CyclinD1, C-Myc, MMP-9, VEGF, TNF-α, IL-6, IL-8, and other pro-inflammatory factors. Continued activation of this pathway can lead to cell canceration (Li, 2009).

**FIGURE 6:**
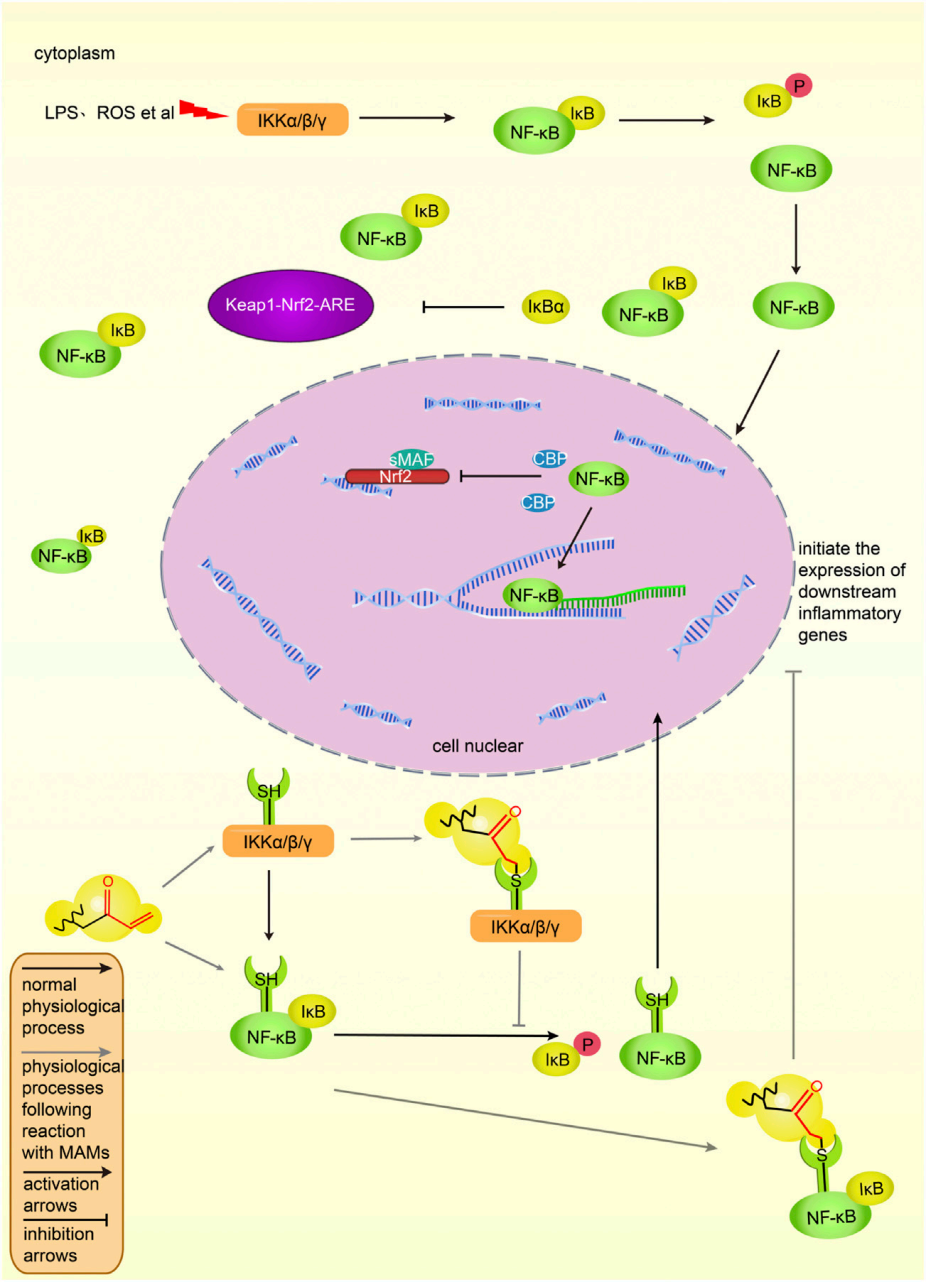
The NF-κB pathway and its intersection action with Keap1-Nrf2-ARE *in vivo*. NF-κB binds to IκB and is in an inactive state in the cytoplasm. Under the stimulation of lipopolysaccharide and ROS, IKK phosphorylates IκB, and NF-κB dissociates from the complex, enters the nucleus, combines with the corresponding DNA sequence, initiates the transcription of inflammatory factors, and induces inflammation. IκB can promote the activation of the Keap1-Nrf2-ARE pathway, and NF-κB can prevent the binding of Nrf2 to CBP and inhibit the transcription of ARE genes. MAMs bind to cysteine residues of the IKK complex or NF-κB, inhibiting the dissociation of NF-κB and IκB, preventing NF-κB binding to inflammatory genes and inhibiting inflammatory factors expression. The black solid line represents the original process of inflammation *in vivo*, and the gray solid line represents the inhibitory process of MAMs on the NF-κB pathway. Normal arrows indicate activation and T-shaped arrows indicate inhibition.

Under physiological conditions, p65 and p50, the two subunits of NF-κB, bind to inhibitory protein IκBα, thereby exist in the cytoplasm and are inactive (Wang et al., 2019). After cells are stimulated by ROS, LPS and other signals, the cascade of signals activates the IKK complex, which phosphorylates and degrades IκBα (Li, 2009). Two subunits, p65 and p50, are activated, move to the nucleus, combine with related inflammatory genes to start the transcription of inflammatory factors and induce inflammation (Li, 2009). Figure 6 shows the NF-κB pathway in activated and inhibited states *in vivo*.

##### 3.2.2.2 Inhibitory pathway

There are many ways to inhibit the NF-κB pathway. The α, β-unsaturated structures of MAMs can directly inhibit IKK by binding to the cysteine residues in the activation loop of IKKβ (Liu and Cao, 2005), thereby preventing IκBα phosphorylation and blocking the NF-κB pathway. For example, physalin A exerts anti-inflammatory activity by targeting Cys59, Cys179, Cys299, Cys370, Cys412, and Cys618 residues in the IKKβ loop (Ji et al., 2012). The main activation signal pathway of IKK is the MAPK pathway. PKC is the upstream activator of MAPK (Xu et al., 2008). The N-terminus of PKC is rich in cysteine residues. Also, the α, β-unsaturated structure may inhibit the NF-κB pathway by binding to cysteine residues of PKC (Lee S. E. et al., 2011). In addition, there are a variety of NF-κB pathway inhibitory pathways. Figure 6 shows the inhibitory effect of MAMs on the NF-κB pathway.

##### 3.2.2.3 Interaction between the Keap1-Nrf2-ARE pathway and the NF-κB pathway

NF-κB p65 can negatively regulate Nrf2, and the negative regulatory protein IκBα of NF-κB can activate the Nrf2 pathway and inhibit the NF-κB pathway (Yu et al., 2011). NF-κB inhibits Keap1-Nrf2-ARE at the transcriptional level: p65 prevents Nrf2 from binding to the ARE and inhibits the Keap1-Nrf2-ARE pathway; in addition, p65 inhibits Nrf2 binding to ARE by promoting the interaction between histone deacetylase three and CBP (Liu et al., 2008).

##### 3.2.2.4 Michael acceptor molecules that inhibit the NF-κB pathway by inhibiting the phosphorylation of IκB-α, p65 or p50

4,2′,5′-trihydroxy-4′-methoxychalcone can inhibit the production of tumor necrosis factor-α (TNF-α) and interleukin-1β (IL-1β). It inhibits the production of inflammatory mediators such as inflammatory factors downstream of NF-κB pathway, COX-2 and iNOS, by inhibiting the phosphorylation of IκBα and nuclear translocation of p65 (Lee et al., 2013). The activation of macrophages caused by LPS can be inhibited by ILQ, ILG, and Liquiritigenin (LQG) (Wang R. et al., 2015). However LQG lacking a MAM structure has the weakest inhibitory effect (Wang R. et al., 2015). ILQ and ILG show better anti-inflammatory effects, which may owe to the long-conjugated structure of chalcone. Among them, ILQ has a larger steric hindrance, so the anti-inflammatory effect of ILG is stronger. ILQ, ILG, and LQG can obviously prevent the phosphorylation and degradation of IκBα (Wang R. et al., 2015), prevent p65 nuclear translocation, downregulate NF-κB transcription and protein expression, and inhibit the production of inflammatory mediators (Zeng et al., 2017). ILG can also inhibit RANKL-stimulated NF-κB expression and nuclear translocation (Zeng et al., 2017).

Salvianolic acid B can inhibit NF-κB phosphorylation to downregulate the NF-κB pathway and reduce inflammation (Li et al., 2021). CEL can reduce the phosphorylation level of IκBα, inactivate p65 and inhibit the NF-κB signaling pathway or interact with NF-κB through p38MAPK to improve the inflammatory response caused by LPS and exert anti-inflammatory effects on retinal pigment epithelial cells (Zhang et al., 2019). It can also reduce the p65 phosphorylation caused by γ-radiation to block NF-κB, reduce excessive inflammation, and increase the survival rate after γ-radiation (Wang et al., 2020). The PP31J can inhibit the NF-κB pathway by reducing the phosphorylation level of IκBα (Xu et al., 2014), increase the level of ROS in tumor cells, cause cycle arrest (Ding et al., 2014) and inhibit cell proliferation.

PYDDT can inhibit the expression of p65 and downregulate the NF-κB pathway in a dose-dependent manner (Zhang et al., 2010). Zerumbone can negatively regulate TLR4, inhibit the phosphorylation of NF-κB/p65, reduce the protein expression level of COX-2, and reduce the production of IL-6 and TNF-α in the tissues of mice with liver injury (Wang et al., 2019). It inhibits the production of inflammatory mediators, controls the state of OS and exerts a hepatoprotective effect.

Sulforaphane can inhibit the phosphorylation of IκBα (Huang et al., 2019), bind to essential sulfhydryl groups involved in NF-κB activation, or interact with GSH or other NF-κB-related redox regulators (such as thioredoxin and Ref-1) to directly inactivate NF-κB and downregulate LPS-induced iNOS, COX-2 and TNF-α (Heiss et al., 2001). ESC can reduce the levels of ROS and p65, downregulate the expression of c-myc, CyclinD1 and caspase 3, regulate the levels of Bax and Bcl-xL in pancreatic cancer cells and induce cancer cell apoptosis (Wang R. et al., 2015). It can also inhibit IκB phosphorylation and transfer p65 from the nucleus to the cytoplasm, block NF-κB binding to DNA and downregulate the expression of anti-apoptotic genes (Li et al., 2019b). Ferulic acid can significantly reduce p65 expression, reduce the level of NF-κB and exert an anti-inflammatory effect (Doss et al., 2016). The structure of Helenalin contains two MAMs structures. It can inhibit the NF-κB pathway by binding to Cys38 of p65 (Widen et al., 2018). Helenalin analogs that do not contain the MAM structure are inactive, confirming that the active center of Helenalin is the structure of MAM (Widen et al., 2018). Andrographolide can covalently bind to Cys62 of p50 through MAR and non-covalently interact with other binding sites to form adducts to inhibit the NF-κB pathway (Nguyen et al., 2015).

##### 3.2.2.5 Michael acceptor molecules that inhibit the NF-κB pathway by regulating other proteins except IκB-α, p65 or p50

Melissoidesin G has the ability to inhibit TNF-α, induced NF-κB transcriptional activation and induce tumor cell apoptosis (Yu, 2007). It has less toxicity and side effects on normal cells, and its anticancer activity is linked to the structure of α-methylene cyclopentanone (Yu, 2007).

Xn can inhibit the overproduction of inflammatory mediators like NO, IL-1β and TNF-α, and inhibit the activation of the NF-κB pathway in microglia induced by LPS (Lee I. S. et al., 2011). Hydroxysafflor yellow A (HSYA) can reduce the levels of IL-1β, TNF-α and NO to inhibit LPS-induced neuronal damage by inhibiting the expression of NF-κB/p65 and iNOS (Miao, 2019). HSYA can also reduce inflammation and protect brain damage caused by cardiac ischemia-reperfusion (Ye and Gao, 2008) through inhibiting the levels of IL-1β, IL-6, TNF-α, COX-2 and iNOS *via* the Bcl-2/Bax and PPAR-γ signaling pathways (Miao, 2019).

α-cyperone can downregulate LPS-induced NF-κB pathway in microglia and inhibit the production of TNF-α, IL-6 and IL-1β, thereby inhibiting inflammation and exerting neuroprotective effects (Huang et al., 2018). The specific mechanism may inhibit NF-κB activation by combining with p65, p38, extracellular signal-regulated kinase (ERK) and c-Jun N-terminal kinase (JNK) and reducing the phosphorylation of mitogen-activated protein kinases (Zhang et al., 2021). 4-hydroxy-3-methoxycinnamaldehyde can reduce the phosphorylation of mitogen-activated protein kinases and reduce the transcriptional activity of NF-κB, AP-1, and NFAT (Akber et al., 2015).

α-cyperone can also upregulate SIRT1, thereby inhibiting NF-κB and NLRP3 signaling pathways to inhibit LPS-induced acute lung injury (Liu X. et al., 2019). N-caffeoyltyramine contains α, β-unsaturated ketones and o-diphenol hydroxyl structure. It is a MAM with relatively strong NF-κB inhibitory activity, which is the main reason that the root bark of *L. chinense* Mill. or *L. barbarum* L. Inhibits the NF-κB pathway (Xie et al., 2014). Costunolide (COS) contains a MAM structure and can alleviate pulmonary fibrosis by modulating NF-κB and TGF-β1/Smad2/Nrf2-NOX4 signaling pathways (Liu B. et al., 2019).

#### 3.2.3 Michael acceptor molecules that function by regulating other proteins

##### 3.2.3.1 Michael acceptor molecules that function by regulating the AKT-Nrf2 signaling pathway

HSYA can reduce the percentage of apoptosis, increase the ratio of Bcl-2/Bax, increase AKT phosphorylation and nuclear translocation of Nrf2, and promote the expression of HO-1. It can exert cardioprotective effects and enhance antioxidant effects by regulating the AKT-Nrf2-HO-1 signaling pathway (Hu et al., 2021). Safflower yellow B has strong antioxidant properties. It enhances antioxidant genes expression through the AKT-Nrf2 pathway, inhibits mitochondrial-related apoptosis and eliminates OS (Ma et al., 2016). α-cyperone can enhance the nuclear translocation of Nrf2 and upregulate HO-1 expression by activating AKT (Ye and Gao, 2008). Crotonaldehyde contains α, β-unsaturated aldehyde structure and has highly activity in inducing the expression of HO-1 *via* the PKC-δ-p38 MAPK-Nrf2-HO-1 pathway and mediating OS in human umbilical vein endothelial cells (Lee S. E. et al., 2011).

##### 3.2.3.2 Michael acceptor molecules that function by activating Nrf2 with unclear upstream components

4,2′,5′-trihydroxy-4′-methoxychalcone can induce HO-1 expression by promoting nuclear translocation of Nrf2 (Lee et al., 2013). The α, β-unsaturated carbonyl structure of isosalipurposide can phosphorylate extracellular signal-regulated kinase 1/2 and AMPK, activate the Nrf2-ARE pathway and induce the expression of phase II detoxification enzymes in hepatocytes (Han et al., 2015). Flavokawains A in Kava can activate Nrf2, increase the expression of antioxidant genes such as GSH and HO-1, and it has a protective effect similar to sulforaphane against H_2_O_2_-induced cell death (Pinner et al., 2016). ILG can activate Nrf2-mediated antioxidant gene expression, regulate ROS, and reduce brain damage caused by cerebral ischemia (Zhang et al., 2019). It can also upregulate the expression of HO-1 through extracellular signal-regulated kinase 1/2 (Zhang et al., 2019). FA can enhance anti-inflammatory activity by activating the AMPK-Nrf2 pathway (Calabrese et al., 2021). COS can regulate Nrf2, promote the nuclear translocation of Nrf2, reduce the level of ROS, restore the balance of thiols, and exert neuroprotective effects against oxidative damage in cells (Peng et al., 2019).

##### 3.2.3.3 Michael acceptor molecules that function by regulating proteins except Keap1-Nrf2-ARE and NF-κB

ESC can inhibit the phosphorylation of STAT3 during macrophage differentiation, downregulate the production of VEGF and TGF-β1 in tumor cells and inhibit tumor growth (Li et al., 2019b). ESC can regulate MEK/ERK and JNK pathways to decrease intracellular GSH levels and can also induce cell apoptosis through MAPK and EGFR/PI3K/AKT pathways (Li et al., 2019a).

Oridonin can upregulate the transcriptional and translational levels of the NQO1 gene and increase QR activity by activating the PI3K pathway and inhibiting the ERK pathway in Hepalclc7 cells (Wang et al., 2017). In addition, oridonin is cytotoxic to human melanoma cells, mouse fibrosarcoma cells, human erythroleukemia cells, human histiocytoma cells, and human promyelocytic leukemia cells (Zuo et al., 2005).

Men et al. (2016) have isolated eight MAMs from the *P. minima* L., which are 3-isopropyl-5-acetoxycyclohexene-2-one-1, physalin G, physalin D, physalin I, physordinose B, isophysalin B, stigmasterol-3-O-β-D-glucopyranoside and 5α-6β-dihydroxyphysalin R. These compounds can induce QR, and the inductive activity is proportional to their ability to bind GSH (Men et al., 2016). Among them, isophysalin B had the strongest QR induced activity, which was higher than the same dose of 4′-bromoflavone (Men et al., 2016).

Xn can regulate other pathways by covalently modifying cysteine residues in proteins, such as partially inhibiting glucose-6-phosphate dehydrogenase, limiting the production of NADPH, and inhibiting tumor growth (Brodziak-Jarosz et al., 2016). The weak electrophilicity of Xn makes it more selective, which enhances the therapeutic potential of Xn (Brodziak-Jarosz et al., 2016). Gambogic acid (GA) is a MAM. The unsaturated ketone in the structure is the functional center of GA anti-proliferation (Wang J. et al., 2015). New gambogic acid, which is structurally similar to GA, induces apoptosis through the mitochondrial pathway and death receptor pathway (Wang et al., 2011). CEL, curcumin (CUR) and rosmarinic acid can target the cysteine-rich domain of Tat, a transactivator of HIV gene transcription, through α, β-unsaturated carbonyl structure (Narayan et al., 2011). It combines with the sulfhydryl group of cysteine, changes the conformation of Tat and affects the interaction of Tat-tar, thereby inhibiting the reverse transcription of HIV (Narayan et al., 2011).

Cinnamaldehyde analogs containing α, β-unsaturated side chains, hydrophobic parts and negative charges can combine with the nucleophilic amino acid side chains of *Vibrio* LuxR through MAR to form an irreversible cinnamaldehyde receptor complex, thereby influencing the binding of LuxR and DNA, regulating the expression of virulence genes of *Vibrio* bacteria, and affecting biofilms formation and protease production. It can also increase the survival rate of the nematode *Caenorhabditis elegans* infected with *Vibrio* anguillarum, *Vibrio* harveyi and *Vibrio* Vulnificus (Brackman et al., 2011).

Fang et al. (2021) found that helenalin inhibits hepatic stellate cell activation by inhibiting miR-200a-mediated PI3K/Akt and NF-κB pathways, thus it can be used as a potential drug for the treatment of liver fibrosis. Dehydrocostus lactone has been proved to inhibit the NF-κB pathway by scavenging ROS, activating Nrf2, and downregulating IKK and JNK, thereby inhibiting differentiation of osteoclasts and treating diseases such as osteoporosis caused by excessive activity of osteoclasts (Lee et al., 2020).

The eupachiilide A, eupachinilide B and eupalinilide G are all MAMs. Experiments have revealed that the three compounds are cytotoxic to triple-negative breast cancer cells (Jiang et al., 2020). Among them, eupachiilide A has the strongest cytotoxicity to triple-negative breast cancer cells. The mechanism of action may be that the electrophilic α, β-unsaturated carbonyl reacts with the nucleophilic residue of the corresponding active site, leading to a series of biological activities (Jiang et al., 2020).

## 4 Discussion

The above elucidated that MAMs can regulate the Keap1-Nrf2-ARE pathway and the NF-κB pathway, thus MAMs can treat diseases related to these two pathways. Keap1-Nrf2-ARE pathway is associated with OS, which in the organism can lead to cell damage, leading to a variety of chronic diseases in the body, including renal ischemia/reperfusion injury, diabetic nephropathy, pancreatitis, hepatitis, lung ischemia-reperfusion injury, *etc.* (Majima et al., 2016; Adelusi et al., 2020; Wang et al., 2020). MAMs can reduce OS by binding to Keap1 and promoting Nrf2 nuclear translocation, which is theoretically effective for these diseases. NF-κB regulates processes such as inflammation, immunity, apoptosis, and cancer development, and is associated with the development of diseases such as colon cancer and neurodegenerative diseases (Seo, 2011; Seo et al., 2019). MAMs can also theoretically be used to treat diseases regulated by the NF-κB pathway. This paper only summarizes the common action pathways of MAMs. Most proteins in the human body contain sulfhydryl groups, such as fructose-1,6-bisphosphate aldolase, pyruvate dehydrogenase, ferrochelatase, *etc.* The former two are involved in the process of glycolysis. MAMs, which can react with sulfhydryl groups, can theoretically participate in most life activities in the human body, regulate the function of proteins, and have a great impact on the body. Drugs developed based on the structure of MAMs are also gradually entering people’s lives. As a third-generation EGFR inhibitor, Osimertinib (Callegari et al., 2018) works by covalently binding to Cys797 of EGFR through the Michael receptor structure, overcoming the drug resistance caused by the first-generation inhibitor. Brutinib (Raedler, 2015) reacts with Cys481 of Bruton’s tyrosine kinase, inhibits the activity of Bruton’s tyrosine kinase, and has good curative effects on chronic lymphoblastic leukemia tumors. However, the current research on MAMs is still insufficient, and the complex mechanism of MAMs action *in vivo* is still worthy of in-depth study in the future. Existing drugs can exert the effect of increasing efficiency and reducing toxicity through structural modification. We can also expand the choice of drugs in combination with antibiotics and add foods mainly containing MAMs to the diet to interfere with diseases.

### 4.1 Structural modification of existing compounds

Many natural medicines have excellent curative effects on intractable diseases. The special and diverse structures of natural products can provide new ideas for drug research and development. MAMs containing α, β-unsaturated ketone structure have good antitumor effects. For example, Ligustrazine has a strong antitumor effect, and its derivatives obtained by introducing α, β-unsaturated carbonyl or α, β-unsaturated imine structures have lower toxicity and stronger antitumor activity (Zha et al., 2017). Salvicine is a compound obtained by introducing a MAM structure into the diterpene quinone compound derived from *Salvia. Prionitis* Hance. It may exert anti-tumor effects through two pathways: depleting GSH by reacting with intracellular GSH, promoting the generation of H_2_O_2_, inducing DNA double bond breakage, mediating the Topo II/DNA complex; modifying the sulfhydryl group of Topo II (Cai et al., 2008). Kakuol has bacteriostatic effect on some plant pathogenic bacteria. After structural modification, it was found that the kakuol derivatives with α, β-unsaturated ketones have antibacterial activity against various phytopathogenic fungi, and the activity is higher than the original compound (Sui, 2017). They can be used as new active components for agricultural prevention and control of plant diseases. Compared with traditional pesticides, they have the advantage of reducing the pollution of land and crops (Sui, 2017). Baldwin (Baldwin et al., 2015) modified the structure of CUR while keeping the carbonyl group and double bonds on both sides, the resulting compound is effective for the treatment of *Mycobacterium tuberculosis* (MTB) and rifampin-resistant MTB. The active center may be the carbonyl group and the double bonds on both sides. Reducing the structure of the double MAMs weakens or even eliminates the inhibitory effect of CUR structural analogs on MTB (Baldwin et al., 2015). 3′,4′,5′,4″-tetramethoxychalcone (isolated from *Spatholobus suberectus* Dunn) has good cytotoxicity and its derivatives removing α, β-unsaturated carbonyl or cyclized chalcone proved to be less cytotoxic to tumor cells, indicating that the structure of MAM may be the active center (Peng et al., 2016).

There are also some toxic natural MAMs. The toxicity of these compounds can be reduced by modifying the structure of the toxic center. Biliatresone, a natural toxin found in *Dysphania glomulifera* and *D. littoralis*, 1,2-diaryl-2-propenone is its Michael structure toxicity center, resulting in high biliary toxicity (Koo et al., 2015). The toxicity of biliatresone can be reduced by structural modification of the toxicity center.

### 4.2 Combined with drugs to treat diseases

H_2_S is a gas signal molecule in the human body, that is, involved in physiological processes such as vasodilation, cell protection, and energy production. H_2_S is produced and released in humans *via* the catalysis of cystathionine β-synthase (CBS) and cystathionine γ lyase (CSE). CBS, CSE, and H_2_S are highly expressed in human malignant osteoblastoma cells. These proteins and small molecules may play a role in promoting the proliferation of tumor cells (Hu et al., 2016). Some scholars (Shatalin et al., 2021) found that H_2_S in *Staphylococcus aureus* and *Pseudomonas aeruginosa* is mainly produced by CSE, which generates H_2_S by using l-cysteine as a substrate. H_2_S mediates the bacterial defense system and promotes bacterial resistance. The MAMs readily react with nucleophilic groups due to the electrophilicity generated by the structure. The sulfhydryl group on l-cysteine and H_2_S in the sulfhydryl state can all react with the MAMs, thereby inhibiting the generation of bacterial drug resistance, enhancing the effect of antibiotics, and increasing the choice of drugs for clinical treatment of drug-resistant bacteria. In addition, many natural MAMs have low toxicity to normal cells, which can be used as a better option in dealing with diseases. The anti-inflammatory effect through the CSE/H_2_S pathway is also a new idea for studying MAMs. Melissoidesin G combined with As_2_O_3_ can induce apoptosis of various leukemia cells, and it can also be used in combination with other chemotherapeutic drugs to enhance the induction of apoptosis (Yu, 2007).

### 4.3 Diet supplement for disease prevention

Excessive ROS speeds up the development of chronic and degenerative diseases like cancer, lung diseases, and problems with the digestive system (Liu et al., 2018). Foods with antioxidants as main ingredients are believed to eliminate ROS and intervene in human diseases with low toxicity and long-term efficacy and have great potential in the treatment of human diseases (Wolf, 2001). Wolf (2001) proposed that the Nrf2-ARE antioxidant pathway is an important pathway to prevent human diseases and the research on this pathway has a huge impact on the treatment of human tumors. MAMs can activate Nrf2, promote ARE transcription and induce the expression of antioxidant enzymes. *C. longa* L. (Peron et al., 2019) and cruciferous plants (Soare et al., 2017) with MAMs as the main components have antioxidant and anti-inflammatory effects. They can be used as one of the choices for dietary intervention in diseases.

### 4.4 The application of Michael acceptor molecules in COVID-19

MAMs also play a significant role as covalent inhibitors in the COVID-19. Covalent inhibitors have greater affinity, more stable binding and stronger effects than non-covalent inhibitors. During the replication cycle of the SARS-CoV-2, PLpro and Mpro proteases are required to cut the proteins synthesized in host cells to form non-structural proteins. Inhibition of PLpro and Mpro can weaken the replication of the virus (Narayanan et al., 2022). Both of them are therapeutic targets of SARS-CoV-2 (Narayanan et al., 2022). The medicines created for Mpro are expected to have good efficacy and broad-spectrum anti-SARS activity since the binding pocket structure of Mpro is highly conservative. Most of the COVID-19 inhibitors, including PF07321332 and Paxlovid, are made for Mpro. The Mpro active site contains a catalytic dyad composed of Cys145 and His41 (Narayanan et al., 2022). Yang et al. (2005) developed the inhibitor N3 through computer aided drug design based on Michael receptor structure. Jin et al. (2020) observed the binding of N3 and Mpro by molecular docking. After kinetic analysis, they found that the vinyl carbon atom of N3 and the sulfhydryl group of Cys145 underwent MAR to form a stable covalent bond and formed multiple hydrogen bonds with the residues in the binding pocket, which helped to stabilize the binding. Subsequently, Jin et al. (2020) demonstrated that N3 exhibited a significant inhibitory effect on SARS-CoV-2 by qRT-PCR experiments. The Michael receptor is one of the main warheads to develop covalent drugs at present, which can help compounds target the Cys145 residue of Mpro, and has been proved to inhibit the activity of Mpro (Ramos-Guzman et al., 2021). The 2-methylene-1-tetralone produced by the metabolism of {2-[((4-hydroxyphenethyl)amino)methyl]-3,4-dihydronaphthalen-1(2H)-one} was covalently bind to Cys145 of Mpro by Michael addition to make it inactive and prevent the replication of the SARS-CoV-2 (Günther et al., 2020).

## 5 Conclusion

MAMs can activate the Keap1-Nrf2-ARE pathway to play an antioxidant role by covalently binding to cysteine residues and can also inhibit the NF-κB pathway to play an anti-inflammatory role. It is effective for a variety of tumors and inflammatory diseases. The Michael structure has the advantages of not being easily off-target and having a long duration of action. It can also be used as a warhead to modify small molecules targeted proteins whose active centers contain cysteine residues. Furthermore, it could be used in conjunction with drugs to fight drug-resistant bacteria and enhance the curative effect. MAMs can also play a role in people’s lives as a dietary source to prevent disease. In short, MAMs is interesting category of compounds with great development potential in medical uses.

## Glossary

ALDOA: aldolase A
ARE: anti-oxidant response element
ATP: adenosine triphosphate
BTK: Bruton tyrosine kinase
CBP: CREB binding protein
CBS: cystathionine β-synthase
CDK2: cyclin-dependent kinases 2
CNKI: China Knowledge Resource Integrated Database
COS: costunolide
COVID-19: coronavirus disease 2019
CSE: cystathionine γ lyase
Cul3: ubiquitin ligase E3
CUR: curcumin
Cys: cysteine
EGFR: epidermal growth factor receptor
ERK: extracellular signal-regulated kinase
ESC: esculetin
GA: gambogic acid
GSH: glutathione
GST: glutathione-S-transferase
HAS: human serum albumin
HCAR2: hydroxycarboxylic acid receptor 2
Hcy: homocysteine
HO-1: heme oxygenase
HSYA: hydroxysafflor yellow A
IgG: immunoglobulin G
IKK: IκB kinase
IL-1β: interleukin-1β
ILG: isoliquiritigenin
ILQ: isoliquiritin
JNK: c-Jun N-terminal kinase
Keap1: Kelch-like ECH-associated protein-1
Keap1-Cul3-Rbx1: E3 ubiquitin ligase complex
LDHA: lactate dehydrogenase A
Lico-E: licochalcone E
LQG: liquiritigenin
MAMs: Michael acceptor molecules
MAR: Michael addition reaction
MTB: *mycobacterium* tuberculosis
NQO1: NAD(P)H: quinone oxidoreductase 1
Nrf2: nuclear factor erythroid 2-related factor 2
OS: oxidative stress
QR: quinone reductase
ROS: reactive oxygen species
SARS-CoV-2: severe acute respiratory syndrome coronavirus 2
SMAF: small Maf
SOD: superoxide dismutase
TNF-α: tumor necrosis factor-α
XN: xanthohumol.
